# Pharmacy education in Romania: alumni perspectives on curriculum relevance and professional readiness

**DOI:** 10.3389/fmed.2025.1678666

**Published:** 2026-01-07

**Authors:** Marius Călin Cherecheș, Hajnal Finta, Aura Rusu

**Affiliations:** 1 Faculty of Pharmacy, George Emil Palade University of Medicine, Pharmacy, Science and Technology, Târgu Mureș, Romania; 2 Faculty of Medicine, George Emil Palade University of Medicine, Pharmacy, Science and Technology, Târgu Mureș, Romania

**Keywords:** alumni feedback, competency-based curriculum, curriculum relevance, graduate competency assessment, health professions education, pharmaceutical workforce readiness, pharmacy education, Romanian pharmacy graduates

## Abstract

**Background:**

Pharmacy education is undergoing global transformation to better align with evolving healthcare demands. However, in Romania, pharmacy curricula remain centrally regulated and standardized, with limited input from graduates or employers.

**Objective:**

This study investigates the perceptions of Romanian pharmacy graduates (2009–2023) regarding the relevance of their curriculum, competency development, and professional preparedness across various employment sectors.

**Methods:**

A mixed-methods, cross-sectional survey was conducted with 473 alumni from all accredited pharmacy faculties in Romania. Quantitative data were analyzed using descriptive statistics, ANOVA, and logistic regression, while qualitative responses underwent thematic content analysis.

**Results:**

Graduates reported moderate overall preparedness (Mean = 3.08/5), with significant variation by institution, age, and job sector. High self-assessed competencies were noted in Pharmacology and Communication, while Management, Regulatory Affairs, and Therapy management were identified as key gaps. Disciplines such as Pharmacology and Clinical Pharmacy were rated highly useful, whereas Physics and Inorganic Chemistry were frequently cited as disproportionately difficult and less applicable. Open-ended responses emphasized the need for greater curricular flexibility, experiential learning, and integration of soft skills and entrepreneurship.

**Conclusion:**

The results highlight a misalignment between standardized pharmacy education and the diverse realities of professional practice. The study advocates for alumni-informed reforms, modular curricula, and the integration of transversal competencies to enhance workforce readiness and sector-specific applicability.

## 1 Introduction

While programs around the globe adapt the academic experience to address the rapidly evolving needs of the healthcare system, there has been significant evolution in pharmacy education in recent years. Once mainly based on pharmacology and the basic sciences, numerous curricula have been redesigned to focus on patient-centred care, interprofessional collaboration, and experiential learning. Care, system, and personal communication skills have been integrated into the pharmacist training curricula, reflecting a dramatic shift from rigid disciplinary boundaries to the dynamic didactic toolbox of interprofessional and interdisciplinary teaching efforts (1–3).

Many PharmD programs in the United States have adopted integrated curricula, and case-based and team-based learning strategies are becoming increasingly common (4–6). To prepare students for the complexities of real-world environments, educational institutions in Canada and Australia have also adopted digital simulations and virtual placement platforms (7). Incorporating communication skills into early clinical exposure in the United Kingdom is now considered best practice (8, 9). In Vietnam and the Middle East, new frameworks are emerging to link the quality of education with measurable student satisfaction and competencies (10–12). These initiatives indicate an increasing awareness that, in contemporary pharmaceutical practice, mere technical ability is not enough. Today, skills such as critical thinking, leadership, adaptability, professional branding, and interpersonal skills are essential for pharmacists to thrive in both clinical and non-clinical roles (13–15). The change in perspective has also prompted studies on the efficacy of curricula, cross-domain credit hour mapping, and graduate readiness assessment (16–18).

In Asia, curricular reforms are increasingly aimed at fostering competencies aligned with international standards, despite local constraints in infrastructure and policy implementation (19). By strongly emphasizing management abilities, job satisfaction, and context-appropriate teaching methods, African nations like Nigeria are also addressing the critical shortages in pharmacy workforce training. International research and healthcare employers have also raised concerns regarding the capacity of pharmacy graduates to interact with patients, work in interdisciplinary teams, and adapt to challenging healthcare settings. Studies from Vietnam and Nigeria support these deficiencies by linking pharmacists’ stress and job dissatisfaction to a lack of preparation in non-clinical domains such as career development, administrative responsibilities, and emotional resilience (19, 20).

Despite regional differences in advancement, a global trend is emerging: pharmacy education is shifting towards learner-centred, competency-driven models designed to prepare graduates for more complex, multidisciplinary professional realities.

In China, recent analyses have identified gaps in the technical and managerial training of pharmacy professionals, particularly in specialized services such as intravenous admixture (21). Similarly, structured reflective learning programs can enhance pharmacy students’ communication, teamwork, and preparedness for working in multidisciplinary healthcare environments, as noted in recent interprofessional education initiatives in Australia (22).

Despite significant curricular changes in pharmacy education, a mismatch remains between what graduates learn in the classroom and the skills required in practical application. Many studies have shown that pharmacy graduates often feel unprepared for key components of professional life, particularly in administrative tasks, decision-making and communication (23, 24).

Also, in Saudi Arabia, only 58% of undergraduate pharmacy students reported that they would choose the same degree again, raising concerns about educational satisfaction and career clarity (25). A national survey, which included more than 700 pharmacy interns in Malaysia, revealed moderate satisfaction with the internship experience. However, it also highlighted issues regarding documentation requirements that were deemed too high and unrealistic performance targets set by the Pharmacy Board (26).

The results draw attention to a common problem: pharmacy school typically excels in providing biomedical knowledge, but does not sufficiently develop the thorough, practical skills required in modern practice. Graduates may thus face challenges during the significant transition from academic environments to professional practice, which can impact job satisfaction, performance, and retention.

Although most studies focus on North America, Western Europe, and specific areas of Asia and the Middle East, international research has increasingly examined the efficacy of pharmacy curricula. Empirical data from Central and Eastern Europe—including Romania, where national-level pharmacy education is highly controlled and standardized—are conspicuously lacking. The geographical disparity in the research limits the global understanding of how various institutional and regulatory settings impact graduates’ preparedness and satisfaction.

Existing studies assessing alumni perspectives generally originate from contexts characterized by greater institutional flexibility and higher curricular autonomy, aligning with the knowledge and skills required in practice, where graduates are often consulted in curriculum revisions (12, 27–29). In contrast, countries like Romania implement uniform national curricula aligned with EU directives (e.g., Directive 2005/36/EC). However, no large-scale, graduate-level evaluation of curricular usefulness has been conducted.

Furthermore, most studies published to date concentrate either on employers’ expectations or on students’ impressions in university, rather than on past assessments by graduates with years of professional experience. Based on experience, this viewpoint is crucial to grasp whether pharmacy education successfully supports long-term professional development and adaptation. The lack of such information from controlled European systems creates a significant blind spot in the global debate on pharmacy education.

Pharmacy education in Romania is regulated under a centralized national system, overseen by the Romanian Agency for Quality Assurance in Higher Education (ARACIS). The ARACIS 2021 criteria for the field of pharmacy require that all academic programs—encompassing important scientific and professional areas, including Pharmaceutical Chemistry, Pharmacognosy, Pharmacology, Toxicology, Pharmaceutical Technology, and Legislation—have a standard curricular structure. Furthermore, these national criteria complement European laws, particularly the EU Directive 2005/36/EC, which establishes minimum training requirements for various sectors, including health professions, across member states.

While it limits institutional flexibility and the integration of new capabilities or localised innovations, the framework ensures curriculum consistency and legal adherence across all pharmacy schools in Romania. To the best of our knowledge, there is currently no official approach in place to methodically gather and combine comments from pharmacy graduates on the value and effectiveness of their university education.

Examining the opinions of pharmacy graduates on the supposed value of their academic background helps to close the gap.

The authors finalised a qualitative study on pharmacists’ professional satisfaction in Romania, and the difficulty of academic curricula was mentioned several times, so this quantitative study naturally followed (30).

.Our study aims to evaluate the perceived relevance and effectiveness of pharmacy education in Romania by analyzing the experiences of graduates from 2009 to 2023. Specifically, it evaluates how effectively the standardized national curriculum prepares graduates for diverse professional roles across various sectors, including community pharmacy, hospital practice, industry, academia, and regulatory affairs. The research investigates graduates’ self-reported levels of preparedness, competency in key domains, and the practical utility of individual disciplines. It also explores sector-specific differences in curriculum perception, identifies perceived gaps between academic training and real-world demands, and gathers alumni recommendations for curricular improvement. Integrating quantitative and qualitative data, the study provides evidence-based insights to inform policy, support curriculum reform, and enhance alignment between pharmacy education and workforce expectations within a centrally regulated European framework.

### 1.1 Study objectives

This study aimed to explore the perceptions of pharmacy graduates regarding the usefulness of academic training for professional practice. The specific objectives were:

(a) To assess the perceived relevance and utility of university-level disciplines in relation to current employment roles.
(b) To identify potential curricular gaps based on graduates' feedback.
(c) To explore the association between employment sector and perceived curricular usefulness.

## 2 Methodology

To comprehensively assess the perceived relevance and effectiveness of pharmacy education in Romania, this study employed a mixed-methods, cross-sectional design targeting graduates from all accredited pharmacy faculties across the country. The methodology was structured to capture both quantitative and qualitative insights into alumni experiences, focusing on their self-reported preparedness, competency development, and the practical utility of curricular components.

**Figure 1:**
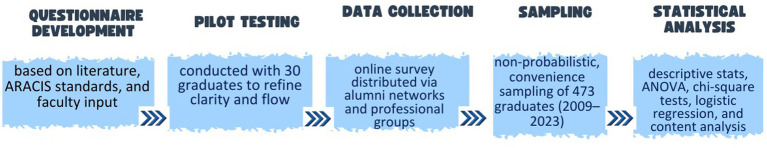
Overview of study design and stages: from questionnaire development to data analysis.

A structured questionnaire was developed based on national academic standards (ARACIS), relevant literature, and expert input from pharmacy educators. The instrument was pilot-tested for clarity and reliability, then disseminated online through alumni networks and professional platforms. This approach enabled the collection of diverse perspectives from 473 pharmacy graduates who completed their studies between 2009 and 2023, offering a robust foundation for evaluating the alignment between academic training and real-world professional demands. The workflow methodology of the study is described in Figure 1.

### 2.1 Questionnaire development and pilot testing

Our research team developed a questionnaire to measure graduates’ satisfaction with their educational experience, the perceived usefulness of the disciplines they studied during their university years, and questions to evaluate professional satisfaction. The part involving graduates’ satisfaction with their education was structured to gather comprehensive data on key aspects of education and professional preparedness.

The study employed a cross-sectional survey design to examine pharmacy graduates’ perceptions of the relevance of university-level academic training to their professional practice.

A structured questionnaire was developed based on the published literature, national ARACIS standards, and informal discussions with faculty members who participated in the design of the pharmacy curricula (Annexe 1 of the Supplementary Materials). The questionnaire included both closed-ended (using Likert scales) and open-ended response questions regarding perceptions of curricular usefulness, training satisfaction, graduate competencies, and potential curricular changes.

Thirty pharmacy graduates comprised the convenience sample for the first questionnaire prototype. Pilot comments led to some items being polished for clarity, and some minor changes were made to improve question flow.

Cronbach’s alpha was calculated to assess the internal consistency of the scales. The items measuring satisfaction with educational competencies produced a Cronbach’s alpha of 0.8439, indicating good reliability. Similarly, the items assessing the perceived usefulness of various disciplines yielded a Cronbach’s alpha of 0.9531, demonstrating excellent internal consistency. The questionnaire’s alpha values, exceeding the 0.70 threshold, confirm its reliability in measuring educational satisfaction and perceived utility, thereby supporting its use.

### 2.2 Data collection

The data were collected through an online questionnaire developed using the Google Forms platform. The electronic survey link was disseminated through several targeted channels to reach pharmacists who graduated after 2009. These channels included Facebook groups dedicated to pharmacists, alumni networks from various universities and several branches of the Chamber of Pharmacists across different regions.

Data collection took place between April 15 and May 12, 2024. Participation was voluntary, and all responses were anonymous to ensure confidentiality and encourage honest feedback. The time required for completion was 10-15 minutes.

As comprehensive national data on pharmacy graduates are not publicly available, the total population size was estimated based on the average admission capacity of pharmacy programs in Romania. We estimate that between 10,000 and 12,000 students graduated between 2009 and 2023. This estimate was used to determine the margin of error for the sample.

The research received approval from the Institutional Review Board (IRB) of George Emil Palade University of Medicine, Pharmacy, Science, and Technology of Târgu Mureș, as indicated in correspondence recorded under number 3049 dated April 12, 2024.

### 2.3 Population and sampling

The target population consisted of alumni from the Faculties of Pharmacy in Romania who graduated between 2009 and 2023. The choice of 2009 as the lower boundary was intentional, as it marks the post-crisis period during which job market instability and changes in the pharmacy market may have influenced the graduates’ transition into professional life and their retrospective evaluation of their training.

A non-probabilistic, convenience sampling strategy was employed. A total of 473 complete and valid responses were collected. The sample size yields a margin of error of ±4.43% at a 95% confidence level for the estimated population.

Although the sampling was not random, the respondent pool included a diverse range of graduation cohorts, employment sectors (community, hospital, industry, regulatory, academic), and job levels, increasing the potential representativeness of the data.

### 2.4 Statistical analysis

Data analysis was performed using Stata/SE 14.0 (StataCorp LLC, 2019). Means, standard deviations, and frequencies were used to describe the characteristics of the respondents and their responses to the survey. Inferential statistics consisted of a one-way ANOVA to compare medians of job category and satisfaction scores, and a chi-square test to determine relationships between categorical variables (e.g., perceived adequacy of curricula and current type of work). Perceived curriculum adequacy and satisfaction with the current job category were also examined for their association via bivariate correlations. A value of *p* < 0.05 was considered to be significant. Open-ended questions were subjected to inductive content analysis. Standard codes and themes reflect graduates’ qualitative recommendations for curriculum enhancement and perceived training deficiencies.

## 3 Results

### 3.1 Demographics of respondents

The study included 473 pharmacy graduates, with data collection restricted explicitly to those who completed their studies between 2009 and 2023. Sociodemographic characteristics of Romanian pharmacy graduates (2009–2023) are presented in Table 1 of the Supplementary materials. Most respondents were female, comprising 88.37%, while males accounted for 11.63%. The gender distribution is consistent with trends typically observed in healthcare professions, particularly in the field of pharmacy. Regarding geographical distribution, 90.27% of respondents reported living in urban areas, with the remaining 9.73% residing in rural areas. Regarding marital status, over half of the participants (55.60%) were married, while 39.75% identified as single. A small percentage of respondents reported being in a domestic partnership (2.11%), divorced (2.11%), or widowed (0.42%). Age distribution data showed that the most significant proportion of participants (52.22%) were between 28 and 35 years old. A considerable segment (26.43%) was younger, falling within the 20–27 age group. Only a small percentage (4.86%) of respondents were over 45.

**Table 1:** Summary statistics for preparedness after graduation by graduation location.

No.	Graduation location	Mean preparedness	SD	*N*	Level 1 (%)	Level 2 (%)	Level 3 (%)	Level 4 (%)	Level 5 (%)
1	UMF Carol Davila	2,9	1,218	80	13,75	26,25	27,5	21,25	11,25
2	UMF Craiova	2,73	0,961	15	6,67	33,33	46,67	6,67	6,67
3	UMF Gr. T. Popa, Iași	2,93	1,163	43	13,95	18,6	37,21	20,93	9,3
4	UMF Iuliu Hațieganu, Cluj-Napoca	3,06	1,054	62	11,29	12,9	38,71	32,26	4,84
5	UMF Victor Babeș, Timișoara	2,89	1,396	27	22,22	14,81	33,33	11,11	18,52
6	UMFST G. E. Palade, Târgu Mureș	3,24	1,101	187	8,56	13,37	36,36	29,41	12,3
7	Universitatea Dunărea de Jos	2,85	1,281	13	23,08	7,69	38,46	23,08	7,69
8	Universitatea Oradea	3,42	1,165	12	8,33	8,33	33,33	33,33	16,67
9	Universitatea Ovidius	2,6	1,429	20	30	20	25	10	15
10	Universitatea Titu Maiorescu	4		1	0	0	0	100	0
11	Universitatea Vasile Goldiș	3,92	1,115	13	0	7,69	38,46	7,69	46,15
	Total	3,08	1,171	473	12,05	16,49	34,88	24,52	12,05

Respondents were well distributed across the period 2009–2023 in terms of graduation year, with the largest cohorts graduating in 2023 (10.78%), 2020 (9.09%), and 2014 (8.88%); this spread allows for a comprehensive understanding of both recent and more established professionals in the field.

Regarding the location of graduation, the majority of respondents (39.53%) graduated from UMFST G. E. Palade in Târgu Mureș, followed by UMF Carol Davila in Bucharest (16.91%) and UMF Iuliu Hațieganu in Cluj-Napoca (13.11%). Participants in our survey represented every university offering pharmacy programs in Romania.

When asked about their highest level of education and specialization, nearly half of the respondents (48.20%) indicated that they had completed only a bachelor’s degree. In comparison, 29.81% had completed a pharmacy residency (Specialist Pharmacist) and 17.55% had pursued a master’s program. A small percentage had completed a doctorate (PhD) (3.38%) or held the title of Senior Pharmacist (“*Farmacist primar*”) (1.06%).

From a professional standpoint, 68.71% of the graduates currently work in pharmacy-related settings, such as community or hospital settings, while 12.05% are employed in the pharmaceutical industry. A smaller segment is active in the public sector or education (3.38%), during residency programs (Pharmacist in training) (6.98%), or other fields (8.88%).

Seniority in the profession varied: 39.96% had less than 5 years of experience, 32.77% had between 5 and 10 years, and 19.87% had between 10 and 15 years. Only 7.40% had more than 15 years of experience in the field.

Regarding their current role, nearly half of the respondents (49.68%) reported working as pharmacists, while 24.95% were chief pharmacists. Other roles included regulatory specialists (6.77%), teaching staff (2.54%), managers (3.17%), and pharmacy owners (1.06%).

### 3.2 Preparedness level after graduation

The respondents were also asked how well-prepared they felt after graduating. Ratings ranged from 1 (not at all prepared) to  5 (very well prepared). The average preparedness score was 3.08 (SD = 1.17), indicating a moderate level of preparedness. Results revealed that 12.05% of the participants perceived their level of preparedness to be very low, 16.49% as low, 34.88% as neutral, 24.52% as high, and 12.05% as very high, with the most students falling into the neutral category at 34.88%.

The preparedness of pharmacy graduates was evaluated across several factors, including their educational institution, age group, and job position and is presented in Table 1. Furthermore,  logistic regression was used to assess the effects of demographic and academic variables on preparedness perceptions. The means of the preparedness scores, as rated by the graduates at the time of graduation, varied among the institutions. Universitatea Vasile Goldiș in Arad, Universitatea Oradea, and UMFST G. E. Palade in Târgu Mureș revealed mean scores of preparedness that were higher than the average, suggesting that graduates originating from these institutions generally felt sufficiently prepared. Also, UMF Iuliu Hațieganu from Cluj-Napoca had a just above average result, which means its graduates’ preparation was assessed as outstanding. In contrast, the lowest mean preparedness score (Mean = 2.60) was reported for Universitatea Ovidius, indicating the lowest perceived readiness among other institutions. A Pearson chi-square test revealed a statistically significant relationship between the institution where students graduated and their perceived preparedness, *χ*^2^(40) = 59.00, *p* = 0.027, indicating that preparedness is inconsistent across institutions.

Preparedness scores varied throughout age groups, as illustrated in Table 2. Graduates aged 46–65 reported the highest mean preparation score of 3.57, indicating that the demographic felt the most prepared. Recent graduates, specifically those between 20 and 27 years old, exhibited the lowest average score (2.83), indicating a diminished sense of preparedness compared to their older counterparts.

**Table 2:** Summary statistics for preparedness after graduation by age group.

Age group	Mean preparedness	SD	N	Level 1 (%)	Level 2 (%)	Level 3 (%)	Level 4 (%)	Level 5 (%)
20–27 years	2,83	1,183	125	16,8	20	35,2	19,2	8,8
28–35 years	3,1	1,138	247	10,53	17	35,63	25,51	11,34
36–45 years	3,27	1,124	78	8,97	12,82	33,33	32,05	12,82
46–65 years	3,57	1,376	23	13,04	4,35	30,43	17,39	34,78
Total	3,08	1,171	473	12,05	16,49	34,88	24,52	12,05

The trend across age groups suggests that increased age and possibly more life or professional experience contribute to a higher sense of preparedness after graduation.

Differences in perceived preparedness were also observed across job positions, as shown in Table 3. Graduates in teaching positions reported the highest mean preparedness score of 3.92, followed closely by pharmacy owners (Mean = 3.80), suggesting that these roles, which may involve extensive application of knowledge, are associated with higher perceived preparedness. In contrast, managers (not chief pharmacists) reported the lowest preparedness score (Mean = 2.67), perhaps due to the different skill sets required in administrative roles.

**Table 3:** Summary of preparedness after graduation by job position.

No.	Job position	Mean preparedness	SD	*N*	Level 1 (%)	Level 2 (%)	Level 3 (%)	Level4 (%)	Level 5 (%)
1	Other position	2,98	1,21	56	16,07	12,5	41,07	17,86	12,5
2	Teaching position	3,92	1,24	12	8,33	0	25	25	41,67
3	Pharmacist	3,04	1,12	235	11,06	17,87	36,17	25,53	9,36
4	Chief pharmacist	3,19	1,15	118	11,02	11,86	38,14	25,42	13,56
5	Manager (non-chief pharmacist)	2,67	1,4	15	26,67	26,67	6,67	33,33	6,67
6	Pharmacy owner	3,8	1,3	5	0	20	20	20	40
7	Regulatory affairs /QA Specialist	2,91	1,25	32	12,5	31,25	21,88	21,88	12,5
	Total	3,08	1,17	473	12,05	16,49	34,88	24,52	12,05

A binary logistic regression analysis was conducted to examine which demographic and educational factors predicted high levels of perceived preparedness. Age group was a significant predictor: graduates aged 36–45 were more than twice as likely to report feeling well prepared compared to those aged 20–27 (OR = 2.33, 95% CI: 1.25–4.36, *p* = 0.008), and those aged 46–65 had even higher odds (OR = 3.80, 95% CI: 1.37–10.51, *p* = 0.010). Graduation institution and gender were included in the model, but were not statistically significant predictors of preparedness. The overall model was statistically significant [LR *χ*² (13) = 23.41, *p* = 0.037], indicating that preparedness perceptions were meaningfully associated with age and, to a lesser extent, other background characteristics.

### 3.3 Competency in key areas

Graduates were asked to rate their level of competency across several key areas, including pharmacology, pharmaceutical compounding, quality control, fitotherapy, management, and communication. The ratings ranged from 1 (indicating low competency) to 5 (indicating high competency). The summary statistics and frequency distributions for each competency area provide insights into which skills graduates feel most and least competent in (Table 4).

**Table 4:** Summary statistics for competency ratings.

No.	Competency area	Mean preparedness	SD	Level 1 (%)	Level 2 (%)	Level 3 (%)	Level4 (%)	Level 5 (%)
1	Pharmacology	3.71	1.02	3.59	8.25	24.31	40.8	23.04
2	Pharmaceutical compounding	3.48	1.16	6.55	14.38	24.95	32.98	21.14
3	Quality Control	3.06	1.2	13.74	16.07	32.98	24.95	12.26
4	Fitotherapy	3.09	1.17	9.94	21.35	31.92	23.68	13.11
5	Management	2.87	1.2	15.01	24.31	28.96	21.99	9.73
6	Communication	3.55	1.18	7.4	10.57	26	31.92	24.1

The pharmacology area had the highest mean competency rating (Mean = 3.71, SD = 1.02), with 40.80% of graduates rating themselves at level 4 and 23.04% at level 5, indicating that most graduates feel moderately to highly competent in pharmacology. Only 3.59% of graduates rated themselves as level 1 (low competency). Graduates rated their pharmaceutical compounding skills as moderately high, with a mean of 3.48 (SD = 1.16). A substantial portion (32.98%) rated themselves at level 4, while 21.14% selected level 5, reflecting a moderate to high perceived competency. Communication skills received a relatively high rating (Mean = 3.55, SD = 1.18), with 31.92% of graduates rating themselves at level 4 and 24.10% at level 5.

Quality Control and Fitotherapy areas had similar mean competency scores, and the largest group (32.98%) rated themselves at level 3 in both cases.

The competency in Management had the lowest mean score (Mean = 2.87, SD = 1.20), with 15.01% of graduates rating themselves at level 1 and 24.31% at level 2.

The data reveal that graduates feel most competent in pharmacology and communication, with high ratings in these areas. On the other hand, management has the lowest average rating, suggesting that there may be an area where additional training or support could be beneficial. Similarly, quality control and fitotherapy skills show moderate ratings, indicating variability in how graduates perceive their competency in these areas.

An analysis of competency ratings across age groups revealed that most competency areas consistently received ratings, regardless of age, with one notable exception. Older graduates, particularly those aged 46–65, reported significantly higher perceived competency in fitotherapy, suggesting that the skill may develop further with professional experience beyond initial training, as indicated in Table 5.

**Table 5:** Summary of competency ratings by age group (oreded as appreared in questionnaire).

No.	Competency area	Age group 20–27	Age group 28–35	Age group 36–45	Age group 46–65	ANOVA F (*p*-value)
1	Pharmacology	3.58	3.8	3.65	3.74	1.46 (0.225)
2	Pharmaceutical compounding	3.34	3.56	3.49	3.3	1.12 (0.340)
3	Quality Control	3.11	3.13	2.81	2.83	1.83 (0.142)
4	Fitotherapy	2.82	3.17	3.21	3.26	**3.13 (0.026)**
5	Management	2.88	2.85	2.86	3.13	0.40 (0.755)
6	Communication	3.44	3.66	3.42	3.39	1.47 (0.221)

Bold values represents statistical significant at *p* < 0.05.

### 3.4 Usefulness of curriculum components

Graduates evaluated the perceived usefulness of the courses included in their pharmacy curriculum, which comprised both core disciplines mandated by the EU Directive and disciplines required by national standards set by the Romanian Agency for Quality Assurance in Higher Education (ARACIS) (31, 32). The data split by job category is presented in Table 6.

**Table 6:** Perceived usefulness of discipline by job category (oreded as appreared in questionnaire).

Discipline	Overall Mean ± SD	Community pharmacy	Pharmaceutical industry	Public sector/academia	Pharmacist in training	Other	*χ* ^2^	*p*-value
Botany	2.44 ± 1.25	2.54 ± 1.27, N = 324	2.03 ± 1.10, N = 57	3.00 ± 1.46, N = 16	2.42 ± 1.20, N = 33	2.04 ± 1.10, N = 42	27.34	0.038
Physics	1.69 ± 0.98	1.60 ± 0.90, N = 324	1.91 ± 1.14, N = 57	2.75 ± 1.48, N = 16	1.91 ± 1.12, N = 32	1.55 ± 0.67, N = 42	43.55	0
Anatomy	3.67 ± 1.18	3.73 ± 1.15, N = 309	3.38 ± 1.30, N = 56	4.00 ± 0.97, N = 16	4.15 ± 0.91, N = 33	3.05 ± 1.20, N = 40	30.04	0.018
Inorganic chemistry	2.09 ± 1.15	2.03 ± 1.14, N = 321	2.25 ± 1.06, N = 57	3.00 ± 1.46, N = 16	2.18 ± 1.13, N = 33	1.90 ± 1.08, N = 42	32.43	0.009
Organic chemistry	2.23 ± 1.23	2.18 ± 1.23, N = 321	2.33 ± 1.20, N = 57	2.94 ± 1.39, N = 16	2.36 ± 1.17, N = 33	2.02 ± 1.17, N = 41	15.78	0.469
Analytical Chemistry	2.96 ± 1.35	2.19 ± 1.24, N = 322	2.88 ± 1.36, N = 56	3.31 ± 1.40, N = 16	2.52 ± 1.23, N = 33	2.38 ± 1.40, N = 42	37.48	0.002
Pharmaceutical chemistry	2.96 ± 1.35	3.30 ± 1.18, N = 320	3.30 ± 1.18, N = 55	3.50 ± 1.15, N = 15	3.80 ± 1.12, N = 31	2.88 ± 1.31, N = 41	14.93	0.53
Biochemistry	2.97 ± 1.32	2.91 ± 1.31, N = 317	3.19 ± 1.39, N = 57	3.50 ± 1.32, N = 16	3.37 ± 1.19, N = 30	2.63 ± 1.24, N = 41	23.58	0.099
Microbiology	3.03 ± 1.31	3.04 ± 1.33, N = 318	3.00 ± 1.28, N = 57	3.63 ± 1.15, N = 16	3.32 ± 1.17, N = 31	2.56 ± 1.25, N = 41	15.65	0.478
Pharmacology	4.15 ± 1.06	4.22 ± 0.99, N = 269	3.68 ± 1.33, N = 53	4.44 ± 0.96, N = 16	4.62 ± 0.73, N = 29	3.80 ± 1.11, N = 35	39.28	0.001
Pharmaceutical Technology	3.60 ± 1.23	3.58 ± 1.24, N = 305	3.58 ± 1.12, N = 50	4.20 ± 1.05, N = 28	4.00 ± 1.08, N = 14	3.28 ± 1.36, N = 39	21.32	0.167
Toxicology	3.46 ± 1.30	3.52 ± 1.28, N = 301	3.04 ± 1.32, N = 55	3.69 ± 1.30, N = 16	4.00 ± 1.16, N = 32	3.05 ± 1.31, N = 38	28.67	0.026
Pharmacognosy	3.17 ± 1.31	3.23 ± 1.31, N = 317	2.56 ± 1.30, N = 57	3.75 ± 1.12, N = 16	3.55 ± 1.18, N = 31	3.07 ± 1.30, N = 42	33.15	0.007
Legislation	3.39 ± 1.33	3.46 ± 1.30, N = 300	2.91 ± 1.33, N = 53	4.13 ± 1.06, N = 15	3.84 ± 1.19, N = 31	2.84 ± 1.37, N = 38	41.84	0
Cell biology	1.76 ± 1.01	1.74 ± 0.99, N = 316	1.69 ± 0.98, N = 55	2.80 ± 1.26, N = 15	1.90 ± 0.98, N = 31	1.48 ± 0.86, N = 42	37.82	0.002
Environmental chemistry	1.70 ± 0.99	1.68 ± 0.97, N = 294	1.55 ± 0.85, N = 53	2.36 ± 1.34, N = 14	2.03 ± 1.27, N = 29	1.60 ± 0.87, N = 40	22.28	0.134
Physical chemistry	1.62 ± 0.96	1.53 ± 0.87, N = 313	1.89 ± 1.17, N = 56	2.81 ± 1.28, N = 16	1.63 ± 0.89, N = 30	1.48 ± 0.82, N = 40	40.64	0.001
Patient communication	3.42 ± 1.38	3.60 ± 1.27, N = 286	2.54 ± 1.43, N = 50	3.60 ± 1.55, N = 15	3.94 ± 1.21, N = 31	2.76 ± 1.46, N = 41	52.52	0
Dermopharmacy	3.07 ± 1.39	3.18 ± 1.34, N = 281	2.31 ± 1.39, N = 51	3.40 ± 1.50, N = 15	3.71 ± 1.08, N = 28	2.68 ± 1.42, N = 40	36.89	0.002
Clinical pharmacy	3.84 ± 1.27	3.91 ± 1.21, N = 293	3.26 ± 1.42, N = 53	4.13 ± 1.20, N = 16	4.52 ± 0.80, N = 33	3.46 ± 1.41, N = 39	34.35	0.005
Pharmacovigilance	3.37 ± 1.30	3.41 ± 1.28, N = 290	2.95 ± 1.10, N = 44	3.57 ± 1.40, N = 14	3.97 ± 1.16, N = 30	3.03 ± 1.51, N = 39	34.38	0.005
Genetics	2.03 ± 1.21	2.01 ± 1.21, N = 298	1.76 ± 0.95, N = 49	2.69 ± 1.54, N = 16	2.50 ± 1.07, N = 30	1.95 ± 1.32, N = 40	26.89	0.043
Informatics	2.34 ± 1.36	2.25 ± 1.32, N = 311	2.42 ± 1.51, N = 55	3.79 ± 1.31, N = 14	2.50 ± 1.11, N = 32	2.26 ± 1.40, N = 42	41.66	0
Pharmaceutical industry	2.52 ± 1.35	2.38 ± 1.25, N = 311	3.00 ± 1.62, N = 53	3.31 ± 1.49, N = 16	2.75 ± 1.39, N = 32	2.46 ± 1.38, N = 41	42.53	0
Management and marketing	2.74 ± 1.40	2.82 ± 1.39, N = 301	2.30 ± 1.34, N = 53	3.12 ± 1.26, N = 16	2.81 ± 1.33, N = 31	2.51 ± 1.53, N = 41	21.02	0.178
Biologics	2.64 ± 1.33	2.68 ± 1.34, N = 278	2.24 ± 1.20, N = 46	3.23 ± 1.30, N = 13	3.03 ± 1.18, N = 29	2.32 ± 1.43, N = 37	22.34	0.133
Medical devices	2.46 ± 1.35	2.43 ± 1.32, N = 260	2.32 ± 1.43, N = 44	3.47 ± 1.51, N = 15	2.87 ± 1.10, N = 23	2.23 ± 1.33, N = 35	38.87	0.001
Research methods	2.15 ± 1.30	2.00 ± 1.23, N = 286	2.24 ± 1.27, N = 49	3.13 ± 1.51, N = 15	2.69 ± 1.40, N = 32	2.36 ± 1.50, N = 39	29.78	0.019
Nutrition	2.73 ± 1.42	2.83 ± 1.44, N = 270	2.17 ± 1.34, N = 48	3.21 ± 1.48, N = 14	3.04 ± 1.11, N = 26	2.35 ± 1.32, N = 37	24.91	0.071

The mandatory disciplines specified by the EU Directive include fundamental courses such as Botany, Anatomy, Pharmacology, and Pharmaceutical Legislation, among others (see the first 14 in Table 6); these courses are core to the pharmacy curriculum across all institutions and are designed to provide a standardized foundation of knowledge. Among these courses, Pharmacology received the highest usefulness rating (Mean = 4.15, SD = 1.06), highlighting its critical role in professional practice. Pharmaceutical Technology (Mean = 3.60, SD = 1.23) and Anatomy (Mean = 3.67, SD = 1.18) were also highly rated, indicating that graduates perceive these subjects as directly applicable to their work. In contrast, courses like Physics (Mean = 1.69, SD = 0.98) and Botany (Mean = 2.44, SD = 1.25) were rated among the least useful mandatory disciplines. These ratings suggest that while these subjects provide foundational scientific knowledge, graduates may find them less directly relevant to the practical aspects of pharmacy.

The additional disciplines required by ARACIS curricula (listed in the last 15 rows of Table 6) received varying usefulness ratings from graduates. Among these, Clinical Pharmacy was perceived as the most relevant (Mean = 3.84, SD = 1.27, Median = 4), highlighting its strong applicability to real-world pharmaceutical practice. Patient Communication (Mean = 3.42, SD = 1.38) and Pharmacovigilance (Mean = 3.37, SD = 1.30) were also highly valued. In contrast, disciplines such as Physical Chemistry (Mean = 1.62, SD = 0.96) and Cell Biology (Mean = 1.76, SD = 1.01) were seen as less valuable in the context of professional practice.

Several disciplines exhibited statistically significant differences in perceived usefulness across job categories (*p* < 0.05). The strongest associations were observed for Patient Communication (*χ*^2^ = 52.52, *p* < 0.001), Pharmaceutical Industry (*χ*^2^ = 42.53, *p* < 0.001), Physics (*χ*^2^ = 43.55, *p* < 0.001), Legislation (*χ*^2^ = 41.84, *p* < 0.001), Informatics (*χ*^2^ = 41.66, *p* < 0.001), and Physical Chemistry (*χ*^2^ = 40.64, *p* = 0.001).

Graduates working in the public sector or academia rated Physics, Physical Chemistry, Inorganic Chemistry, Legislation, Cell Biology, Medical Devices, and Informatics significantly higher than the overall mean. Those in the pharmaceutical industry rated Patient Communication, Dermopharmacy, Clinical Pharmacy, Pharmacovigilance, Legislation, and Management & Marketing lower than average. Pharmacists in training gave notably higher ratings to Anatomy, Pharmacology, Toxicology, Clinical Pharmacy, and Legislation.

A one-way ANOVA was conducted to explore differences in the perceived usefulness of pharmacy disciplines across various graduation locations. The analysis revealed significant differences for several disciplines. Disciplines such as Environmental Chemistry (*F* = 4.77, *p* < 0.001) and Patient Communication (*F* = 3.38, *p* < 0.001) showed the most notable variations in usefulness ratings between locations. Other disciplines, including Botany, Pharmaceutical Industry, Marketing and Management, and Informatics, also displayed significant differences, suggesting that the institutional context, including curricula and teaching methodologies, may influence perceptions of usefulness.

The one-way ANOVA analysis revealed significant differences in the perceived usefulness of disciplines across job categories for multiple areas, as per Table 7.

**Table 7:** The one-way ANOVA analysis- perceived usefulness of disciplines across job categories for multiple areas.

Discipline	*F*-value	*p*-value	Highest mean group	Mean	Lowest mean group	Mean
Botany	3.97	0.0035	Public Sector/Education	3.00	Pharmaceutical Industry	2.04
Physics	7.17	0	Public Sector/Education	2.75	Pharmacy	1.59
Anatomy	5.79	0.0001	Pharmacist in training	4.15	Other	3.05
Inorganic chemistry	3.39	0.0095	Public Sector/Education	3.00	Other	1.90
Analytical chemistry	6.15	0.0001	Public Sector/Education	3.31	Pharmacy	2.19
Microbiology	2.56	0.0379	Public Sector/Education	3.62	Other	2.56
Pharmacology	5.84	0.0001	Pharmacist in training	4.62	Other	3.80
Legislation	5.93	0.0001	Public Sector/Education	4.13	Other	2.84

The results highlight the varying perspectives on the usefulness of specific disciplines based on current job categories, with certain disciplines, such as Physics and Pharmacology, showing particularly significant discrepancies. Such findings underscore the importance of tailoring educational curricula to align with the demands and expectations of specific career paths.

The Spearman correlation analysis reveals relationships among pharmacy disciplines based on graduates’ perceived usefulness. We classify correlations as follows: above 0.70 is considered strong, 0.50 to 0.69 is moderate, and 0.30 to 0.49 is low; this helps identify clusters of closely interconnected disciplines and those that are more distinct. Several disciplines show strong correlations, highlighting the integrated nature of foundational sciences in the pharmacy curriculum. Inorganic Chemistry and Organic Chemistry exhibit a strong correlation (*ρ* = 0.8798, *p* < 0.05), suggesting that graduates perceive these subjects as interrelated. Similarly, Analytical Chemistry correlates strongly with Inorganic Chemistry (*ρ* = 0.7661, *p* < 0.05) and Organic Chemistry (*ρ* = 0.7792, *p* < 0.05).

The strong correlations in chemical sciences suggest a cohesive understanding due to their foundational role in pharmacological studies. Biochemistry and Microbiology also exhibit a strong correlation (*ρ* = 0.7256, *p* < 0.05), implying that graduates recognize the interdependence of these fields in understanding biological systems and microbial interactions, which are crucial for immunology, pathology, and pharmaceutical research.

Moderate correlations are observed across disciplines, suggesting a perceived relationship among them, although not as strong as in the chemical sciences. For example, Pharmaceutical Technology correlates with Toxicology (*ρ* = 0.5048, *p* < 0.05) and Pharmacology (*ρ* = 0.5929, *p* < 0.05), indicating a link between formulation techniques and drug safety and efficacy. Furthermore, Pharmacognosy, which focuses on medicinal plants, shows moderate correlations with Toxicology (*ρ* = 0.5654, *p* < 0.05) and Pharmacology (*ρ* = 0.5523, *p* < 0.05), highlighting the relationship between natural substances and their therapeutic applications. Legislation, which addresses the legal aspects of pharmacy, correlates moderately with clinical and toxicological fields, such as Pharmacology (*ρ* = 0.5704, *p* < 0.05) and Toxicology (*ρ* = 0.5976, *p* < 0.05), indicating that graduates recognize the value of regulatory knowledge in clinical practice and drug safety.

### 3.5 Relationship between perceptions on curriculum adequacy or practical applicability of various learning activities and current job category

The analysis examined whether graduates’ perceptions of curriculum adequacy varied by job category after graduation or according to graduates’ current job category. The Chi-square test results indicate no statistically significant relationship between these variables [Pearson chi2 (16) = 21.75, *p* = 0.151; Pearson chi2 (16) = 16.23, *p* = 0.437]. Respondents were asked to evaluate their perceived usefulness to explore the effectiveness of various learning activities. The results showed that certain activities, such as laboratories, seminars, and problem-based learning, received high ratings for their applicability. In contrast, online activities and role-playing exercises had higher proportions of “No Usefulness” responses. The Chi-square tests revealed that most learning activities did not exhibit statistically significant associations with job category. However, two exceptions were identified: Courses [chi2 (16) = 25.98, *p* = 0.05] and Pharmacy Practice [chi2 (16) = 51.91, *p* < 0.001]. The obtained results suggest that graduates working in different job categories may perceive these activities differently, potentially reflecting variations in professional demands across sectors.

### 3.6 Perception of disproportion between course difficulty and usefulness

Graduates were asked to name up to three courses they perceived as having a disproportionate balance between difficulty and practical utility, as shown in Table 8. The results indicated that 47.15% of respondents identified three courses, while 27.06% did not list any. An ANOVA analysis revealed significant differences in these perceptions across universities [*F* (10, 462) = 3.35, *p* = 0.0003], suggesting that institutional variations may affect how students evaluate the difficulty and relevance of specific courses. Post-hoc Tukey tests identified that graduates from UMF Carol Davila Bucharest and UMF Iuliu Hațieganu Cluj-Napoca reported significantly more problematic courses than their counterparts from universities such as Oradea University, Vasile Goldiș Arad University, and UMF Victor Babeș Timișoara, who reported fewer concerns. The obtained results suggest that differences in curriculum structure or pedagogical approaches may influence students’ experiences and perceptions of coursework.

**Table 8:** Frequencies by which disciplines were mentioned as disproportionately difficult compared with usefulness.

No.	Discipline	Total frequency	Total percentage (%)
1	Inorganic chemistry	105	12.12
2	Physical chemistry	88	10.16
3	Physics	85	9.82
4	Botany	81	9.35
5	Organic chemistry	78	9.01
6	Analytical chemistry	65	7.51
7	Pharmaceutical chemistry	57	6.58
8	IT/Mathematics/statistics	49	5.66
9	Biochemistry	42	4.85
10	Other	35	4.04
11	Pharmacognosy	30	3.46
12	Cell biology	23	2.66
13	Pharmaceutical technology	19	2.19
14	Genetics	15	1.73
15	Pharmaceutical industry	14	1.62
16	Management/marketing	10	1.15
17	Physiology/pathophysiology	9	1.04
18	Drug analysis	9	1.04
19	Pharmacology	9	1.04
20	Toxicology	9	1.04
21	Physical education	7	0.81
22	Foreign languages	7	0.81
23	Clinical laboratory	6	0.69
24	Legislation	5	0.58
25	Microbiology	4	0.46
26	Hygiene	3	0.35
27	Medical emergencies	2	0.23

The table above illustrates the problematic courses that were most frequently referred to. The prevalence of chemistry and physics subjects suggests that they are complex and less relevant to their future jobs. The observed trend strongly highlights the need for new academic programs to adapt theoretical instructional content to the applied needs of the profession.

Further analyses examined whether the perception of disproportionate course difficulty varied based on graduation year and job position. Although a slight trend was observed, with more recent graduates (particularly from 2020 to 2021) reporting a higher number of problematic courses, the ANOVA test did not reveal significant differences [*F* (14, 458) = 1.32, *p* = 0.1908]. A Chi-square test revealed a marginally significant association between job category and perceived course disproportion [χ^2^ (100) = 124.29, *p* = 0.050]. Community and hospital pharmacy graduates most frequently cited Inorganic Chemistry (57.9%), Physics (82.1%), and Organic Chemistry (69.2%) as disproportionate courses. Those in pharmaceutical industry roles predominantly mentioned Analytical Chemistry (23.1%) and Biochemistry (15.4%), while public sector and regulatory professionals expressed concerns about Management/Marketing (12.5%) and Pharmaceutical Technology (18.2%). These findings suggest that job sector influences course perceptions, potentially reflecting the degree to which specific subjects are applied in different career paths.

### 3.7 Perception of disciplines the respondents considered necessary for the pharmacy curriculum

The survey included an open-ended question (Q4.9) in which respondents were asked to indicate the disciplines they considered necessary for the pharmacy curriculum. Responses were coded and grouped into thematic categories to facilitate analysis.

Table 9 presents the frequency and percentage of respondents who suggested including or further deepening specific courses in the curriculum. Pharmacovigilance, Pharmacy Practice, Communication, Therapy Management and Counselling, Clinical Pharmacy and Clinical Studies and Business and Entrepreneurship, and Legislation and Regulatory Affairs were among the most frequently mentioned areas, highlighting the perceived need for enhanced training.

**Table 9:** Frequency of indicated courses as needed in university curricula.

No.	Course category	Frequency	Percentage (%)
1	Pharmacovigilance	45	11.6%
2	Pharmacy practice	44	11.4%
3	Communication	76	19.6%
4	Clinical pharmacy and clinical studies	48	12.4%
5	Business and entrepreneurship	44	11.3%
6	Legislation	28	7.2%
7	Regulatory affairs	34	8.8%
8	Therapy management and counselling	71	18.3%
9	Anatomy and physiology	44	11.3%
10	Pharmaceutical industry	18	4.6%
11	IT and digital health	19	4.9%
12	Nutrition and food supplements	18	4.6%
13	Pharmacoeconomics and public health	8	2.1%
14	Other	95	24.5%

A chi-square test was performed to examine whether the likelihood of mentioning specific disciplines varied across different graduation years. The results indicated that respondents from specific cohorts more frequently mentioned certain courses. Notably, the Pharmaceutical Industry course category showed a statistically significant association with graduation year (*p* = 0.023), indicating that more recent graduates were more likely to perceive a need for additional training in this area. Similarly, Regulatory Affairs (*p* = 0.094) and Pharmacovigilance (*p* = 0.088) showed marginal significance, indicating possible trends that warrant further exploration.

A factor analysis was conducted to identify latent structures within the indicated course categories, and it identified three thematic clusters: (1) Regulatory/Clinical/Industry, (2) Legislation/Business/IT, and (3) Therapy Management and Communication. ANOVA revealed that Factor 1 was strongly associated with employment in the pharmaceutical industry (*p* < 0.001), while Factor 3 was linked to pharmacy practice roles (*p* = 0.0103) and varied by graduation location (*p* = 0.0128).

Logistic regression revealed significant associations between job sector and the perceived relevance of discipline. Graduates from the pharmaceutical industry were more likely to value Pharmacovigilance, Clinical Pharmacy, and Regulatory Affairs (OR = 0.56, 0.34, and 0.37, respectively). Public sector and academic professionals emphasized Pharmacoeconomics (OR = 0.65), while those in pharmacy practice highlighted Therapy Management and Communication (OR = 0.86). Odds ratios reflect decreased likelihood compared to the reference group (community pharmacy), indicating sector-specific needs.

## 4 Discussions

The results of our study reveal some essential dimensions of the perceived utility of pharmacy education among Romanian graduates. Although the core scientific subjects were considered relevant, discrepancies were perceived between the competencies focused on during academic training and the practical needs of the workforce. Among the reported intermediate degrees of fit between preparation and practice were communication, administrative work, and working across disciplines. These findings support the broader claim that pharmacy education, particularly in centrally regulated systems, often fails to provide a workforce that is as staff-ready and flexible as needed. The information found serves as a reference for comparison with the international literature and highlights the policy and curricular implications of the results discussed later.

### 4.1 Interpretation of findings in the national and international context

The results of our study reveal a persistent and multifaceted gap between the theoretical orientation of Romanian pharmacy education and the practical demands of contemporary pharmaceutical roles. Many respondents expressed uncertainty about their readiness to meet the complex expectations of real-world practice.

Most of the pharmacy graduates in Romania perceived the learning of sciences in the way they were initially studying such subjects as Pharmacology and Clinical Pharmacy. However, many were uncertain about their ability to meet challenging professional expectations. Communication with patients was most frequently perceived as well prepared for, particularly by community and hospital-based pharmacists; however, other aspects were seen as less well supported through academic training. The respondents reported a lack of exposure to practical decision-making, clinical case scenarios, and pharmacovigilance. They applied features of therapy management, domains that are increasingly central to modern pharmacy but remain underrepresented in traditional curricula.

The results highlight a gap between theoretical education and practical skills in contemporary pharmacy. The gap between theoretical education and practical skills in pharmacy and health professions education is not unique to Romania. International literature consistently highlights the limitations of discipline-based, content-heavy curricula in preparing graduates for the dynamic, interdisciplinary nature of healthcare environments.

For instance, Katajavuori et al. (33) found that pharmacy students in Finland struggled to integrate theoretical knowledge into practice without sufficient exposure to real-world contexts. Their study emphasized the importance of early and sustained practical training in bridging this divide, noting that even short placements significantly enhanced students’ motivation and understanding of academic content.

Kleinheksel et al. (34) argue that experiential learning must be accompanied by structured reflection to be genuinely effective. Drawing on Kolb’s experiential learning theory, they demonstrate that reflection enables learners to internalize and apply knowledge more meaningfully, thereby transforming abstract concepts into actionable skills. In the Romanian context, pharmacy education remains predominantly theoretical and lacks structured opportunities for reflective learning.

Cornish et at. (35) further reinforces this perspective by framing professional development as a process of identity formation that requires the integration of knowledge, action, and reflection. He critiques traditional stage-based models of learning for failing to account for the contextual and relational aspects of skill acquisition. In pharmacy education, this means that students must not only learn scientific facts but also develop the ability to navigate complex interpersonal, ethical, and organizational challenges—capacities that are best cultivated through practice-based and reflective pedagogies.

The sense of unease is amplified in international literature, suggesting that historical, discipline-based curricula may not produce quality, practice-ready graduates needed (12, 36).

The Romanian graduates’ call for more applied content, including pharmacovigilance, communication, and entrepreneurship, aligns with these international findings. Their feedback highlights the need for curricular reform that extends beyond content delivery to encompass experiential learning, reflective practice, and sector-specific skill development. Without such changes, pharmacy programs risk producing graduates who are scientifically knowledgeable but professionally underprepared.

#### 4.1.1 Perceived usefulness of disciplines

Graduates provided distinct evaluations of the perceived usefulness of individual academic disciplines. Subjects such as Pharmacology (Mean = 4.15), Clinical Pharmacy (3.84), and Anatomy (3.67) were rated as most relevant to day-to-day professional responsibilities, especially by those working in patient-facing sectors. In contrast, disciplines including Physics (1.69), Physical Chemistry (1.62), Cell Biology (1.76), and Botany (2.03) received low utility scores, often being described as overly abstract or disconnected from real-world pharmaceutical tasks.

These results suggest a potential conflict between the scientific foundations of pharmacy training and the day-to-day demands of pharmacy practice. A similar study found that although graduates valued the basic disciplines for conceptual understanding, they expressed concerns about their preparedness for community or clinical pharmacy, especially when the courses lacked practical application or real-life relevance (16).

The perceived usefulness of a discipline is not only shaped by its content but also by how it is taught and contextualized. Braxton et al. (37) introduced the concept of “affinity disciplines,” noting that fields with more flexible epistemological structures tend to adopt student-centred pedagogies that enhance perceived relevance. In contrast, rigid, theory-heavy disciplines may struggle to demonstrate their practical value unless explicitly linked to real-world applications.

Similarly, a previously cited study documented that graduates in India rated therapeutics and clinical-oriented subjects as most valuable in their current roles. At the same time, chemistry-heavy disciplines were perceived as less applicable, especially by those not pursuing academic careers (36).

The link between practical relevance and positive course evaluations is not unique to Romania; it is a common phenomenon. A study showed that students in Qatar who experienced Canadian-accredited curricula perceived courses with clinical components as more valuable and applicable to real-life pharmacy roles (38).

The international comparisons support the interpretation that perceived usefulness is shaped by content and its alignment with job realities. In the Romanian context, the lower scores assigned to disciplines such as Physics and Botany indicate a need to revisit how these subjects are taught and contextualized, possibly through interdisciplinary links or applied modules that clarify their relevance within contemporary pharmaceutical care.

The paper of Sin and Soares (39) argues that disciplinary differences in teaching approaches—especially between “hard” sciences and more applied fields—can influence how students perceive the utility of what they learn. In pharmacy, where both scientific rigour and patient-centred care are essential, bridging this divide is critical. Without pedagogical strategies that connect theoretical instruction to practical outcomes, even essential subjects may be undervalued by both students and graduates.

#### 4.1.2 Preparedness after graduation

Graduates provided mixed assessments of how well their academic training prepared them for real-world professional responsibilities. Approximately 47% considered themselves sufficiently prepared, 39% reported feeling only partially prepared, and 14% felt inadequately prepared; these proportions suggest that nearly half of the respondents entered the workforce uncertain about their readiness.

The identified ambivalence may reflect the theoretical nature of much of the curriculum, which, although conceptually robust, may not fully address the practical realities of contemporary pharmacy roles.

Similar concerns have been documented internationally. For instance, Watson et al. (40) highlight that preparedness in pharmacy must extend beyond technical knowledge to include resilience, adaptability, and interdisciplinary collaboration—skills that are rarely emphasized in traditional curricula.

In Qatar, it was reported that although pharmacy programs emphasized content mastery, graduates often struggled with real-world integration due to a lack of structured transitional support (11). Another study found that Australian students benefited significantly from experiential strategies such as mock interviews and structured reflections, which enhanced their confidence during the transition to employment (41). Similar perceptions have been observed in Sweden, where authors reported that while graduates valued their academic training, many felt only partially prepared for workplace challenges due to a lack of exposure to applied contexts and practice-oriented learning (29). The Romanian findings reflect a broader trend: even when knowledge acquisition is substantial, perceived preparedness may remain moderate if the curriculum lacks applied training components or real-world contextualization. Enhancing experiential learning formats—such as simulations, interdisciplinary projects, and structured internships—could significantly improve graduates’ confidence and readiness for professional practice.

#### 4.1.3 Competency levels by domain

Graduates were asked to rate their current level of competency across several key domains of pharmacy practice. The highest levels of self-reported confidence were noted in Pharmacology (Mean = 3.71), Communication (3.55), and Pharmaceutical Compounding (3.48). In contrast, domains such as Management (2.87), Quality Control (3.06), and Fitotherapy (3.09) received lower ratings, indicating a more modest perception of readiness in these areas.

These patterns suggest that competencies with strong representation in the traditional curriculum, such as pharmacological knowledge and communication in patient contexts, are more confidently retained and applied. The relatively high confidence in communication skills is particularly notable given common assumptions about the lack of emphasis on soft skills in Eastern European pharmacy education.

Conversely, the low self-assessed scores in management echo global concerns about insufficient preparation in administrative and business-oriented competencies. A multi-school study in Poland confirmed that pharmacy students often struggle with professional identity and strategic thinking unless exposed to simulation-based and practice-integrated learning environments (42). Similarly, a study in Iran emphasized the limited perceived importance of business and entrepreneurship skills among students, reflecting gaps in formal instruction on leadership and innovation (43).

The importance of clearly defined and measurable competencies is also emphasized in a scoping review by Batt et al., (44) which highlights the need for competency frameworks that are aligned with real-world practice. Their findings suggest that without such alignment, graduates may feel confident in theoretical knowledge but underprepared for practical responsibilities.

The European Society of Clinical Pharmacy (ESCP) has identified interprofessional collaboration, patient-centred care, and chronic disease management as critical areas where pharmacy graduates often lack sufficient training. These gaps reflect the need for curricula to evolve in response to the expanding scope of pharmacy practice (45).

Cultural competency is another domain where graduates report feeling underprepared. A qualitative study published by Robinson-Barella et al. (46) found that while students recognize the importance of cultural humility, they often lack the experiential learning opportunities necessary to develop these skills. The findings highlight a broader challenge in integrating soft skills and context-sensitive competencies into pharmacy education.

The perception of moderate preparedness is also linked to the availability of post-graduate support and continuous learning resources. A recent national study in Romania found that institutional support and opportunities for ongoing training have a significant impact on pharmacists’ confidence and long-term professional satisfaction (47).

Quality control and fitotherapy were perceived as moderately relevant, with marked differences based on professional role. Older graduates reported significantly higher competence in fitotherapy, suggesting that some of these skills may be acquired through professional experience rather than formal education.

Overall, these findings illustrate a persistent divide between foundational scientific training and managerial or sector-specific readiness. International research recommends early integration of applied modules and real-world simulations to address this imbalance and support confidence across the full spectrum of pharmacy competencies.

#### 4.1.4 Curriculum gaps – open-ended responses

Our study reveals a persistent and multifaceted gap between the theoretical orientation of Romanian pharmacy education and the practical demands of contemporary pharmaceutical roles. Although graduates reported a solid grounding in scientific subjects, such as Pharmacology and Clinical Pharmacy, many felt inadequately prepared to handle the complex demands of real-world professional settings. In the open-ended section of the questionnaire, graduates identified multiple areas they considered underrepresented or missing in the formal pharmacy curriculum. Their suggestions reflect a desire for improved alignment between academic training and the practical demands of current pharmaceutical roles. Among the most frequently mentioned additions were Pharmacovigilance, Therapy Management, and Regulatory Affairs. Graduates also emphasized the need for content related to Entrepreneurship, Business Administration, and Communication in clinical contexts, particularly in managing patient expectations or coordinating with physicians. The curriculum does not provide a systematic structure for hands-on learning or reflective engagement. As a result, students often struggle to translate theoretical concepts into practical applications.

These proposals are consistent with international findings. In Lebanon, it was reported that pharmacovigilance training remained fragmented and insufficiently applied in pharmacy programs, leaving graduates underprepared to handle adverse drug reaction monitoring and risk communication (48). Similarly, it was found that many U. S. A. pharmacy curricula lacked formal modules on entrepreneurship or innovation management, despite the growing demand for leadership and business competencies in pharmaceutical settings (49).

The need for structured exposure to the healthcare business is also reflected in research, which advocates for integrating financial, strategic, and operational topics into the pharmacy curriculum (14). Their study highlights how simulation-based exercises and health system modules improve student readiness for complex and multidisciplinary environments.

Studies by Katajavuori et al. (33) emphasize the importance of early and sustained practical training, while Kleinheksel et al. (34) highlight the role of structured reflection in transforming abstract concepts into actionable skills. Cornish (35) further argues that professional development requires integrating knowledge, action, and reflection—capacities best cultivated through practice-based learning.

Romanian graduates echoed these concerns, calling for more applied content, including pharmacovigilance, communication, and entrepreneurship. Their feedback highlights the need for curricular reform that extends beyond content delivery to include experiential learning, reflective practice, and sector-specific skill development.

Importantly, Romanian graduates did not frame these needs as criticisms of existing courses but rather as requests for complementary content that could enhance the practical value of their education. The consistency of these responses suggests an emerging consensus that the curriculum must evolve beyond technical and scientific mastery to include managerial, clinical, and regulatory competencies essential to modern pharmacy practice. Without such changes, pharmacy programs risk producing graduates who are scientifically knowledgeable but professionally underprepared for the realities of modern healthcare environments.

#### 4.1.5 Curriculum perception vs. employment sector

Graduate responses revealed clear differences in how the curriculum was perceived across employment sectors. While the national pharmacy program follows a standardized, generalist structure, its real-world applicability appears to vary significantly depending on the respondent’s current professional context.

Graduates working in community or hospital pharmacies tended to rate the curriculum as moderately relevant but cited insufficient training in therapy management, patient-centred decision-making, and interdisciplinary coordination. These concerns align with findings from Kosovo, where a study reported that pharmacists in clinical practice viewed traditional curricula as overly theoretical and disconnected from frontline needs, calling for a greater integration of clinical reasoning and applied pharmacotherapy (50).

Graduates in industry or clinical roles expressed concerns about limited exposure to regulatory and administrative content. These patterns align with national findings of a previous research, which showed that early career satisfaction among Romanian pharmacy graduates is strongly influenced by how well academic training aligns with sector-specific job requirements (51).

Brown et al. (52) found that while good teaching predicted course satisfaction, the development of generic graduate skills had a limited influence on employment outcomes, suggesting that sector-specific competencies may be more critical to perceived curriculum relevance.

Pharmaceutical industry professionals reported a substantially weaker alignment between academic preparation and job requirements. Respondents highlighted gaps in training for regulatory strategy, project coordination, and commercialization. Although these roles are increasingly common, many graduates indicated they had to learn relevant skills on the job or through separate post-graduate programs.

By contrast, graduates in academic or public sector roles expressed greater satisfaction with the curriculum. For these respondents, disciplines such as Analytical Chemistry, Legislation, and Research Methodology were considered more relevant. A comparable trend was observed in Canada, where a study found that students aiming for careers in academia or policy tended to perceive the traditional curriculum more favorably than those pursuing direct patient care or industry roles (53).

Teichler (54) also found that graduates in public service and academic roles across Europe and Japan were more likely to perceive their education as relevant to their work, compared to those in private sector roles.

These patterns are consistent with regional observations. In a multi-country study across 18 Arab states, it was found that job satisfaction varied considerably depending on the pharmacy sector, with the highest levels reported among those working in academia and industry, and the lowest among community pharmacists. These sector-based differences also shaped how pharmacists evaluated the relevance of their academic preparation (55).

To address these disparities, Billett (56) advocates for a shift toward work-integrated education (WIE) and work-integrated learning (WIL), which embed real-world experiences into academic programs. He argues that employability should be understood not just as job readiness, but as the capacity to adapt and grow across a career; this approach requires curricula to be responsive to the evolving demands of different sectors.

These sector-based disparities reinforce the limitations of a uniform curricular model. While consistency is essential for accreditation and comparability, the diversity of pharmacy career pathways increasingly demands curricular differentiation. The evidences support a shift toward modular or track-based systems, enabling students to tailor specific aspects of their training to better align with their intended career trajectories.

#### 4.1.6 Disproportion between course difficulty and usefulness

One of the most explicit expressions of dissatisfaction among pharmacy graduates concerned the perceived mismatch between the academic effort required by specific courses and their utility in real-world professional practice. Nearly half of all respondents (47.15%) could name three courses they felt were disproportionately difficult relative to their usefulness.

Courses most frequently cited included Inorganic Chemistry, Physical Chemistry, Physics, and Botany—subjects with a strong theoretical foundation but limited perceived applicability, especially in clinical or commercial pharmacy settings. The frustration was significantly more pronounced among graduates in community and hospital pharmacy roles, who emphasised the lack of direct applicability of these subjects to their daily activities.

Comparable apprehensions have been expressed on a global scale. According to Baumann-Birkbeck et al., (57) pharmacy education necessitates the attainment of competencies through clinical placements or wet laboratory experience, encompassing pharmaceutical knowledge, professional skills, and attitudes for healthcare delivery across various settings. Similarly, Vogeser et al. (58) in Germany argued that competency-based reclassification of theoretical subjects led to greater curricular acceptance and reduced frustration among students by clarifying the applied purpose of difficult content. The challenge appears to stem not from the difficulty of scientific content itself, but from the lack of contextual and applied instructional approaches. When students do not clearly understand how complex theoretical knowledge supports practical decision-making, it risks becoming a cognitive burden rather than an asset. Pedagogical innovations, such as vertical integration of disciplines and scenario-based instruction, may offer viable solutions for improving engagement and long-term retention of foundational knowledge.

#### 4.1.7 Suggested disciplines for inclusion

In addition to assessing the usefulness of current curricular elements, the study invited graduates to suggest new or enhanced areas for the pharmacy curriculum. In the open-ended section of the questionnaire, graduates offered suggestions for disciplines or thematic areas that they believed should be integrated into the pharmacy curriculum. Their proposals revealed a shared perception that specific competencies essential for contemporary pharmaceutical practice were underrepresented or missing entirely. Thus, the need for curriculum reform that better equips graduates for evolving professional roles was highlighted.

Among the most frequently cited were pharmacovigilance, therapy management, and regulatory affairs—topics associated with patient safety and sector-specific regulatory compliance. Communication training, particularly in the areas of clinical interaction and patient counselling, was also emphasized as an area needing further development. Additionally, many respondents expressed the desire for structured entrepreneurship and pharmacy business management training, highlighting the growing importance of private-sector roles and operational responsibilities in pharmacy careers.

These suggestions align closely with reform discussions in other international contexts. In a Scandinavian curriculum review, Kvarnström et al. (59) emphasized the need to embed pharmacovigilance and clinical reasoning early in pharmacy education to support safe therapeutic decision-making. In North Africa, Jara et al. (60) identified a curricular gap in regulatory and health policy topics, advocating for their inclusion to reflect pharmacists’ expanding roles in policy and market authorization. Furthermore, Kheir et al. (61) reported that introducing entrepreneurship modules in pharmacy programs across the MENA region significantly improved graduates’ perceived readiness for independent practice and pharmacy ownership.

These suggestions align with student perspectives in other educational systems. For example, Atkinson (62) found that pharmacy students in the UK consistently called for greater emphasis on communication, clinical reasoning, and entrepreneurial training skills, which are often considered essential for modern pharmacy practice but are poorly represented in standard curricula.

What stands out in the Romanian data is the consistency with which these themes appeared across different sectors and graduation years. Respondents did not advocate for eliminating existing content, but rather for diversifying and contextualizing the curriculum through relevant, applied content. These findings support a shift toward more flexible and modular program structures that can accommodate emerging roles in healthcare, regulation, and pharmaceutical entrepreneurship.

### 4.2 Implications for policy and curriculum reform

The data presented in our study suggest that the current Romanian pharmacy curriculum, although structurally consistent and legally aligned with EU standards, lacks the responsiveness and contextual adaptability needed to prepare graduates for contemporary pharmaceutical roles. Several policy and curricular reforms should be considered to enhance both relevance and graduate satisfaction (Table 10).

**Table 10:** Policy and curriculum reform recommendations.

No.	Recommendation area	Key actions
1	Systematic alumni feedback mechanisms	Implement structured alumni surveys and tracer studies; integrate feedback into accreditation and curriculum development
2	Curricular flexibility	Adopt modular or track-based curriculum structures that allow specialisation in clinical, industrial, or regulatory domains
3	Integration of transversal competencies	Embed communication, leadership, project management, entrepreneurship, and digital health skills across all years
4	Experiential and practice-based learning	Expand simulations, case-based learning, interdisciplinary projects, and real-world placements
5	Inter-institutional innovation and collaboration	Support pilot reform projects across universities, sharing best practices and recognising graduate outcomes as quality indicators

First, there is a clear need to establish systematic alumni feedback mechanisms. Graduate insights are currently absent from curricular evaluation and accreditation procedures. By integrating structured alumni surveys and tracer studies into the national quality assurance framework, institutions could gain vital data on the real-world applicability of their programs. Such practices are already embedded in pharmacy education systems in Canada and the UK, where alumni data inform continuous program development (63, 64).

Second, curricular flexibility must be increased to accommodate diverse professional trajectories. A track-based or modular structure would allow students to specialize in clinical setup, industrial, or regulatory pathways, as seen in several reformed programs internationally; this aspect would also enable better alignment between student interests, labour market needs, and course content.

Third, transversal competencies such as communication, leadership, project management, and professional branding should be further developed and integrated across all years of study. The competencies are not merely supplementary but foundational for effective interdisciplinary collaboration and career adaptability. Incorporating longitudinal skill development, supported by simulation and experiential learning, is essential.

Finally, inter-institutional collaboration may facilitate pilot curricular reform projects, allowing universities to innovate within the constraints of national regulation. The national academic framework could accommodate these experiments by explicitly recognizing alumni outcomes, employer feedback, and skill-based indicators as valid markers of educational quality.

The study supports a shift towards a more dynamic, feedback-informed, and competence-oriented model of pharmacy education in Romania. While adhering to core EU directives, local adaptation is crucial to ensure that graduates are scientifically trained and professionally empowered to meet the evolving demands of the pharmaceutical field.

### 4.3 Alignment and divergence with international trends

The study confirms global trends in pharmacy education by highlighting graduates’ strong competencies in pharmacology and communication, consistent with findings from countries like Qatar, Australia, and Sweden (22, 27, 29). However, it also reveals notable divergences: Romanian graduates report significant gaps in management, regulatory affairs, and therapy decision-making—areas that are increasingly emphasised in international curricula.

Unlike more flexible systems in Canada or the UK, Romania’s centrally regulated, uniform curriculum limits adaptation to diverse career paths, contributing to lower perceived preparedness among those in industry and community pharmacy. Additionally, while international programs integrate emerging topics such as entrepreneurship, digital health, and pharmacovigilance, Romanian alumni identified these as underrepresented, underscoring the need for curricular modernisation to align with evolving global standards (62–64).

**Figure 2:**
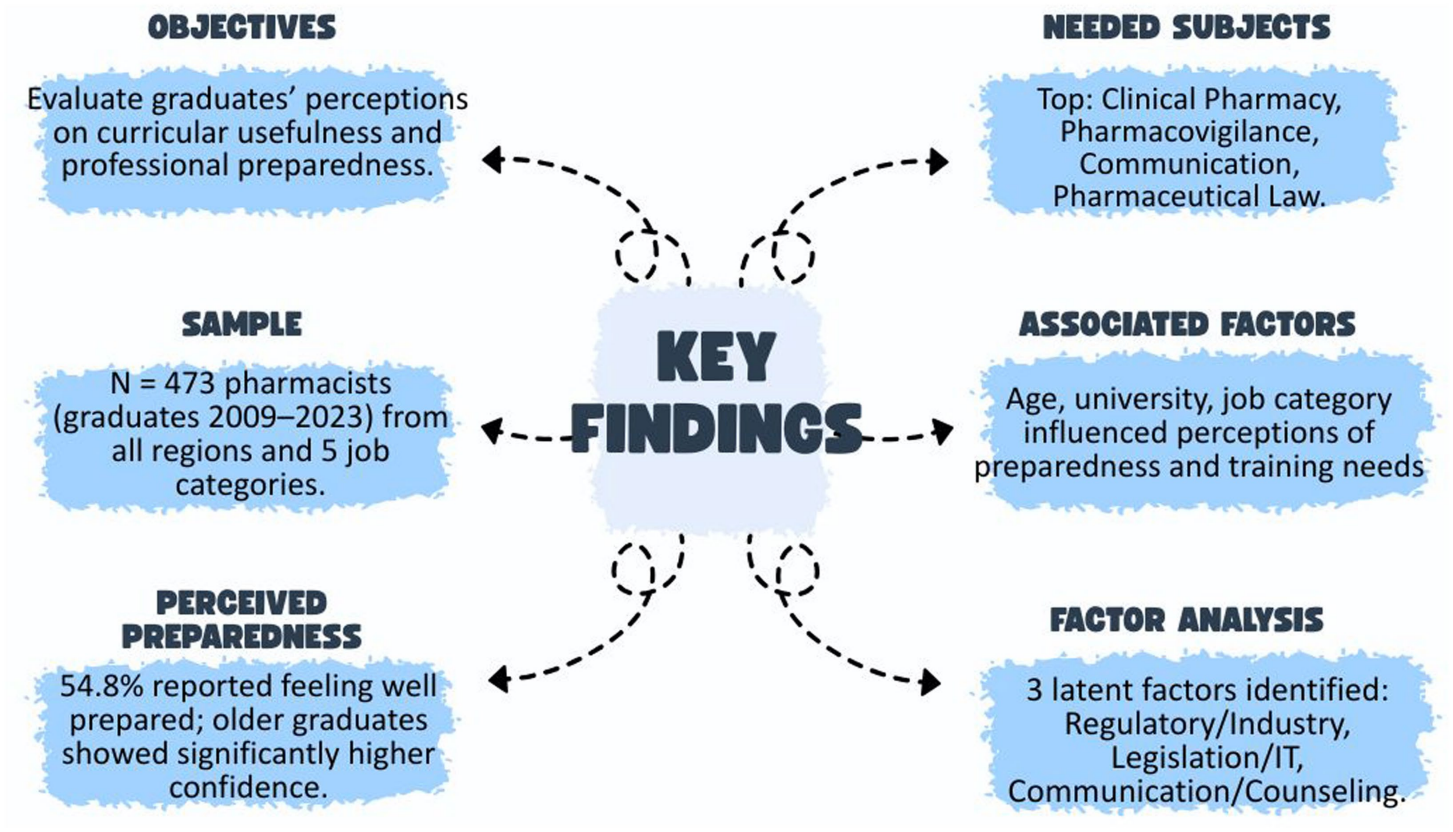
Summary of key findings from the Romanian pharmacy graduates survey (2009–2023). The figure combines quantitative data and insights to highlight gaps between curricula and workforce needs requirements.

A graphical summary of the main findings is presented in Figure 2, highlighting the key insights regarding perceived preparedness, discipline-specific needs, and influencing factors.

### 4.4 Limitations of the study

Several limitations should be acknowledged when interpreting the results of the present study. First, the research employed a cross-sectional design based on retrospective self-reporting, which limits the ability to establish causal relationships between educational experience and professional outcomes. The findings reflect graduate perceptions at a single point in time and may be influenced by memory bias or individual career trajectories.

Second, evaluating curriculum utility and competency development relied exclusively on self-assessment. While subjective insights are valuable, they are also prone to bias, particularly underestimating or overestimating specific abilities depending on the respondent’s current role, confidence level, job satisfaction, or professional environment.

Third, the sampling strategy was based on voluntary participation and convenience sampling. As a result, the sample cannot be considered statistically representative of all pharmacy graduates in Romania. It is possible that individuals with extreme opinions—whether positive or negative—were more likely to respond, potentially skewing the data toward more polarised perspectives. Also, the sample may not statistically represent the entire population of Romanian pharmacy graduates from 2009 to 2023, particularly those who are less active in online professional communities.

Fourth, the temporal distance between graduation and survey participation varied across respondents. Respondents graduated between 2009 and 2023, so their experiences and perceptions may differ significantly based on the duration of their studies and how the job market or curriculum evolved. The variability may have influenced perceptions of curricular relevance, especially as the labour market and professional roles evolve.

Fifth, some groups (e.g., academia, regulatory affairs) are underrepresented, limiting sector-specific generalizability, although the sample includes respondents from various employment sectors.

Sixth, the study did not incorporate perspectives from employers or academic faculty, which limits the triangulation of results. Including these stakeholder views in future research would provide a more comprehensive understanding of curriculum effectiveness and alignment with workforce expectations.

Ultimately, the study focused exclusively on the perspectives of graduate students. While their input is crucial for understanding the long-term impact of pharmacy education, complementary views from educators, employers, and current students would provide a more holistic picture of curricular effectiveness.

## 5 Conclusion

Our research comprehensively assesses how Romanian pharmacy graduates in various settings evaluate their professional skills concerning their training. Data demonstrate a significant divergence between the content emphasis of a standardized curriculum and the applicable skills in modern pharmacy practice.

The perceived relevance of the curriculum differed significantly by employment setting, with graduates working in industry and pharmacy practice considering the curriculum less applicable than those working in academia or regulatory positions. Such variability underscores the inadequacy of a one-size-fits-all curriculum and highlights the importance of greater flexibility and maturity in pharmacy education.

Alumni showed particular interest in the introduction of additional areas of content, such as Pharmacovigilance, Regulatory Affairs, Entrepreneurship, and Therapy Management. These rankings align with global trends toward practice-integrated, skill-based, and feedback-informed curricula.

Our study supports the implementation of alumni feedback mechanisms, modular and track-based structures with curricula for such systems, and the integration of transversal competencies into training at all levels. To adequately prepare students for the real world, pharmacy programs must produce graduates who are knowledgeable, flexible, confident, and capable of fulfilling the diverse roles they are expected to fill. Academic education must therefore reflect the constantly changing professional terrain.

Based on the obtained results, several targeted actions are recommended to enhance the relevance and effectiveness of pharmacy education in Romania:

(a) National education authorities and universities should implement structured alumni feedback mechanisms, such as periodic tracer studies, to systematically gather insights from graduates across sectors;
(b) The current standardized curriculum would benefit from increased flexibility through modular or track-based pathways, allowing students to specialize in clinical, industrial, or regulatory domains;
(c) Transversal competencies—including communication, leadership, entrepreneurship, and digital health—should be integrated longitudinally across the curriculum to better prepare graduates for evolving professional roles;
(d) Experiential learning components such as case-based simulations, interdisciplinary projects, and real-world placements should be expanded to bridge the gap between theory and practice;
(e) Future research should include longitudinal studies that track graduate outcomes over time, as well as comparative analyses involving employer perspectives and international benchmarks, to inform evidence-based policy and curricular innovation.

The study marks the first comprehensive effort in Romania to systematically assess pharmacy graduates’ perceptions of curricular relevance and professional preparedness within a nationally standardized educational framework. The research fills a critical gap in the European pharmacy education literature by capturing alumni insights from over a decade of cohorts and across multiple employment sectors. It provides empirical evidence to inform policy and curriculum reform.

## Data Availability

The raw data supporting the conclusions of this article will be made available by the authors, without undue reservation.
